# Image Processing for Public Health Surveillance of Tobacco Point-of-Sale Advertising: Machine Learning–Based Methodology

**DOI:** 10.2196/24408

**Published:** 2021-08-27

**Authors:** Ned English, Andrew Anesetti-Rothermel, Chang Zhao, Andrew Latterner, Adam F Benson, Peter Herman, Sherry Emery, Jordan Schneider, Shyanika W Rose, Minal Patel, Barbara A Schillo

**Affiliations:** 1 NORC at the University of Chicago Chicago, IL United States; 2 Center for Tobacco Products, US Food and Drug Administration Silver Spring, MD United States; 3 Truth Initiative Schroeder Institute Washington, DC United States; 4 Department of Behavioral Science College of Medicine and the Center for Health Equity Transformation University of Kentucky Lexington, KY United States

**Keywords:** machine learning, image classification, convolutional neural network, object detection, crowdsourcing, tobacco point of sale, public health surveillance

## Abstract

**Background:**

With a rapidly evolving tobacco retail environment, it is increasingly necessary to understand the point-of-sale (POS) advertising environment as part of tobacco surveillance and control. Advances in machine learning and image processing suggest the ability for more efficient and nuanced data capture than previously available.

**Objective:**

The study aims to use machine learning algorithms to discover the presence of tobacco advertising in photographs of tobacco POS advertising and their location in the photograph.

**Methods:**

We first collected images of the interiors of tobacco retailers in West Virginia and the District of Columbia during 2016 and 2018. The clearest photographs were selected and used to create a training and test data set. We then used a pretrained image classification network model, Inception V3, to discover the presence of tobacco logos and a unified object detection system, You Only Look Once V3, to identify logo locations.

**Results:**

Our model was successful in identifying the presence of advertising within images, with a classification accuracy of over 75% for 8 of the 42 brands. Discovering the location of logos within a given photograph was more challenging because of the relatively small training data set, resulting in a mean average precision score of 0.72 and an intersection over union score of 0.62.

**Conclusions:**

Our research provides preliminary evidence for a novel methodological approach that tobacco researchers and other public health practitioners can apply in the collection and processing of data for tobacco or other POS surveillance efforts. The resulting surveillance information can inform policy adoption, implementation, and enforcement. Limitations notwithstanding, our analysis shows the promise of using machine learning as part of a suite of tools to understand the tobacco retail environment, make policy recommendations, and design public health interventions at the municipal or other jurisdictional scale.

## Introduction

### Background

Tobacco point-of-sale (POS) advertising, consisting of signs, displays, and other promotional materials, is considered a very deliberate and effective marketing strategy [1]. The 1998 Master Settlement Agreement restricted tobacco advertising in general, raising the importance of POS advertising as one of the only remaining channels tobacco companies could use to directly reach consumers. In 2018, POS advertising represented the largest category of advertising expenditure for cigarette manufacturers in the United States [2]. Importantly, research has consistently demonstrated the direct influence of tobacco POS advertising on tobacco use [1,3]. Exposure to tobacco POS advertising has been positively associated with the urge to smoke and negatively associated with cessation among adult smokers [3]. Exposure to tobacco POS advertising is positively associated with susceptibility, initiation, and current tobacco use among youth and never smokers [4-6]. In communities where there is more tobacco POS advertising, tobacco use is higher; conversely, smoking rates are lower in communities that have adopted policies restricting tobacco POS advertising [7]. Furthermore, the effects of tobacco POS advertising contribute to disparities in smoking and tobacco-related diseases, as potentially vulnerable populations are often explicitly targeted. Key examples include greater tobacco retailer density in communities of color and pervasive POS advertising of menthol cigarettes in lower-income African American communities [8-11].

The regulation of tobacco POS advertising varies substantially across communities and states within the United States [12]. Surveillance of tobacco POS advertising is important for assessing compliance with local, state, and federal regulations; informing evidence-based policy making; understanding industry behavior; and identifying factors contributing to ongoing disparities in tobacco use and tobacco-related disease burden [13]. At present, store audits represent the most common and rigorous approach to measuring tobacco POS advertisements. In general, researchers recommend a store audit to involve the careful observation of advertisements and retail spaces, speaking with store clerks, and manually photographing and annotating the advertisements present in brick-and-mortar tobacco retailers [1,13]. Such audits require substantial resources, training, and time; thus, they may be difficult to conduct in underresourced communities. For example, a 2014 study surveying 48 states found that the majority of surveillance work at the local level was conducted by volunteer staff [14]. Furthermore, at the time of the study, only slightly more than half (54%) of the surveyed states reported conducting surveillance activities in the past several years. Among those conducting surveillance, only 19% reported that these activities were routine, reinforcing the challenges of consistent data collection via traditional surveillance methods and the importance of leveraging new technology and strategies. Technological advancements would thus be beneficial for improving the depth and breadth of in-store surveillance.

Over the past decade, 2 technological innovations have advanced sufficiently to offer the possibility of a more efficient, less resource-intensive approach for measuring POS advertising at scale. Specifically, the combination of crowdsourcing and machine learning offers a promising strategy to automate the store auditing process and allow researchers to gain insights into actual advertisements. Machine learning is a general term to describe a collection of related computer tasks, including image classification and object detection—key elements in identifying tobacco brands in photographs of store environments. One advantage of machine learning is that it eliminates the burden of manual review and coding and carries the potential for major cost and time savings for research projects. Although both traditional surveillance activities and crowdsourcing efforts have been used to collect data from tobacco retailers, machine learning is yet to be applied to improve digital photograph extraction [14-17].

### Objectives

The primary aim of this study is to assess the feasibility of using machine learning approaches to identify and quantify information accurately in tobacco POS advertising. The proposed approach has 2 components. First, we make use of a large collection of interior photographs of tobacco retailers in West Virginia and Washington, DC. Second, we apply image classification and object detection to identify the brand, number, and size of tobacco advertisements within digital photographs. With respect to image classification, we aim to identify individual tobacco brands in the archive of photographs. We aim to accurately determine the location of brand-specific advertising images in individual photographs by using object detection. By accurately classifying branded images and automatically detecting the location of branded imagery in a retail environment, our approach can provide a practical alternative to in-person POS store audits. In addition, being able to capture enhanced contextual information about the POS environment may provide insights into efforts to combat ongoing efforts from the tobacco industry to use their advertisements to target vulnerable populations.

## Methods

### Data Collection

Camera glasses were used by field staff to collect interior photographs from 410 tobacco retailers across 37 counties in West Virginia from September 2013 to August 2014, the majority of which were in 6 counties with full coverage of all stores. A total of 86,683 digital photographs of tobacco product counters and advertisements were collected. These photographs were then used in the first machine learning task, which attempted to classify specific tobacco brands within the photos. We also collected an additional 13,264 photographs from 82 tobacco retailers in Washington, DC, during the fall of 2018 for the second machine learning task, which focused on identifying the location of advertisements within each photograph.

### Data Cleaning

We manually sorted the photographs into training and validation sets for purposes of training a neural network to classify the retailer photographs by brand. In so doing, we removed the images that contained no advertising or had unclear information, leaving 0.8% (694/86,683) photographs deemed most useful for brand detection analysis between West Virginia and Washington, DC. Although 0.8% (694/86,683) seems low, our cameras took photographs every 1 second, meaning most did not contain any tobacco POS advertising. Once the 694 clearest photographs were selected, they were manually sorted by which brands were contained within them. Finally, we created a training set of 70% (486/694) of the photographs and a testing/validation set of 30% (208/694) of the photographs for classification.

To detect the location of advertisements within each photograph, only photographs with Marlboro advertisements were ultimately used, but from a larger pool of photographs, as not all brands needed to be clear. We decided only to attempt to detect the location of Marlboro advertisements because they were by far the most common brand, giving us the largest amount of data on which to train our model. A sample of the 843 clearest Marlboro photos across Washington, DC, and West Virginia were sorted into training (589/843, 69.9% photographs) and testing sets (254/843, 30.1% photographs).

### Classifying the Presence of Brands in Photographs

Once the images in the training and test sets were manually classified, we used the Python TensorFlow and Keras libraries through the Jupyter Notebooks platform to recreate the manual brand classification with machine learning [18,19]. Fundamentally, our model analyzed each image, resulting in scored and predicted probabilities that a given image contained specific tobacco brands.

Several steps were taken to speed up learning and minimize the computation time owing to the computationally expensive nature of image classification models. First, all processes were executed on a Linux server hosted by Amazon Web Services. Second, we used a pretrained image classification network, Inception V3, which has already been trained to classify millions of labeled images in the *ImageNet* repository [20-22]. Our brand classification model extends Inception V3 to categorize tobacco brands by training a new classification layer on top of its existing architecture, allowing us to successfully classify brands with considerably fewer images than would otherwise be necessary.

After importing the pretrained Inception V3 classification network, we built a deep learning neural network to classify whether the images contained specific tobacco brands. A deep learning network can recognize features in the images that are associated with a targeted brand, including the colors, shapes, or patterns in a given logo. Specifically, we used TensorFlow to configure our computer graphics processing units before training our neural network using Keras. To prevent overfitting and make the most of the 486 photographs in our training set, we configured several random transformations so that our model would never see the same exact picture twice. We randomly applied zooms, shearing, and horizontal transformations to our training set and processed 50 images per batch at a resolution of 299×299 pixels. Once the neural network was trained, we generated the predicted probabilities that each image contained a brand of interest. In our analysis, an image was classified as containing the logo of the brand if the probability was ≥0.5, but other cutoffs could have been chosen.

### Discovering Object Location Within Images

**Figure 1:**
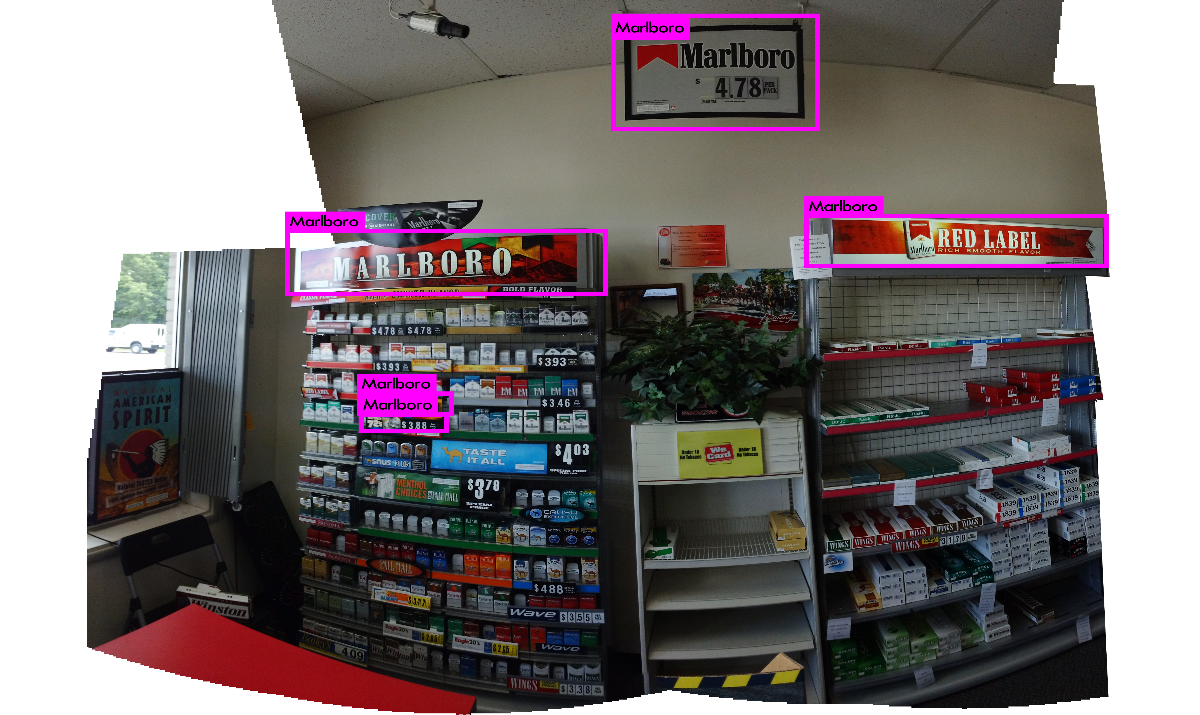
Example image from a tobacco point of sale with YOLO (You Only Look Once) bounding boxes.

Beyond identifying the presence of specific brands, our second goal is to discover their locations in a given photograph to inform their positioning and density in an individual store. Doing so required a different technology than the Inception V3 classification network described earlier, which was designed to discover logos anywhere in an image. We trained YOLO (You Only Look Once) V3, a state-of-the-art, real-time unified object detection system to detect tobacco advertisements within images, as illustrated in Figure 1 [23]. Compared with region-proposal-based convolutional neural networks (eg, R-CNN [region-based convolutional neural networks], fast R-CNN, and faster R-CNN), YOLO V3 uses a single convolutional neural network optimized end to end to full images to simultaneously predict multiple bounding boxes and their class probabilities. By being informed of the global context of the image, YOLO V3 has shown the ability to predict fewer false positives in background image areas where objects are not present [24].

As part of our process, we first used the open-source Visual Object Tagging Tool to draw bounding boxes around each Marlboro advertisement within images and generate related annotations for training [25]. We then trained YOLO V3 using a pretrained, publicly available Darknet-53 model on ImageNet to perform customized object detection [24,26]. Anchor boxes, predefined boxes to improve speed and efficiency in detection of typical objects of interest, were recomputed using K-means clustering with intersection over union (IOU) as the distance measure. We stopped training at the 10,000th batch, where the training loss levels off. To reduce overfitting and generalization error, we tested the network using weights from alternative stopping points generated earlier via the testing data set.

We then evaluated the model accuracy for location detection using testing images based on two metrics: the mean average precision (mAP) and IOU. The mAP is a composite accuracy indicator that ranges from 0 to 1 and accounts for both precision and recall, which is computed as the area under the precision-recall curve. An mAP score of 1 indicates that 100% of the model’s predictions are correct and that 100% of the truth objects are detected by the model. The IOU measures the extent to which the prediction overlaps with the ground truth, which is given by the ratio of the area of intersection and area of union of the predicted bounding box and ground-truth bounding box.

## Results

### Overview

We present our results following our 2 primary research questions, as outlined earlier. First, we describe the success of identifying individual tobacco brands in our set of photographs. Second, we detail how we determined the location of such images in individual stores. We then discuss our findings and their implications in the *Discussion* and *Conclusions* sections.

### Brand Detection

**Figure 2:**
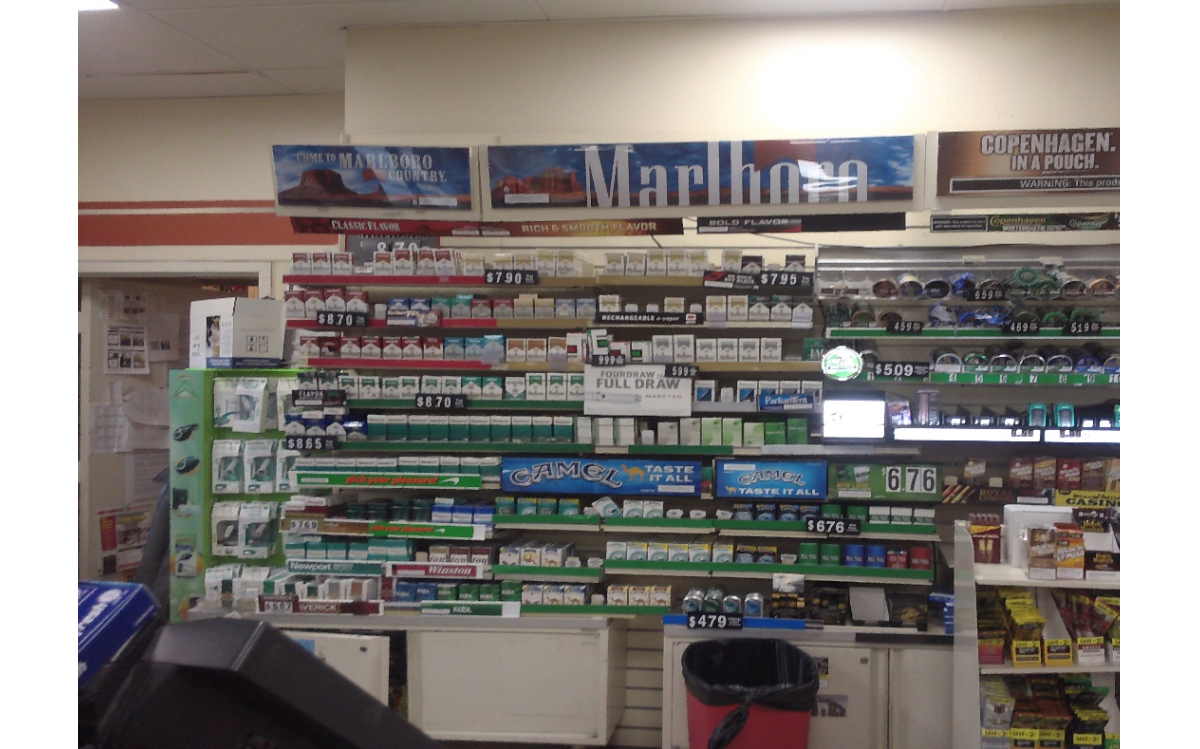
Panoramic image of a tobacco point of sale—view 1.

**Figure 3:**
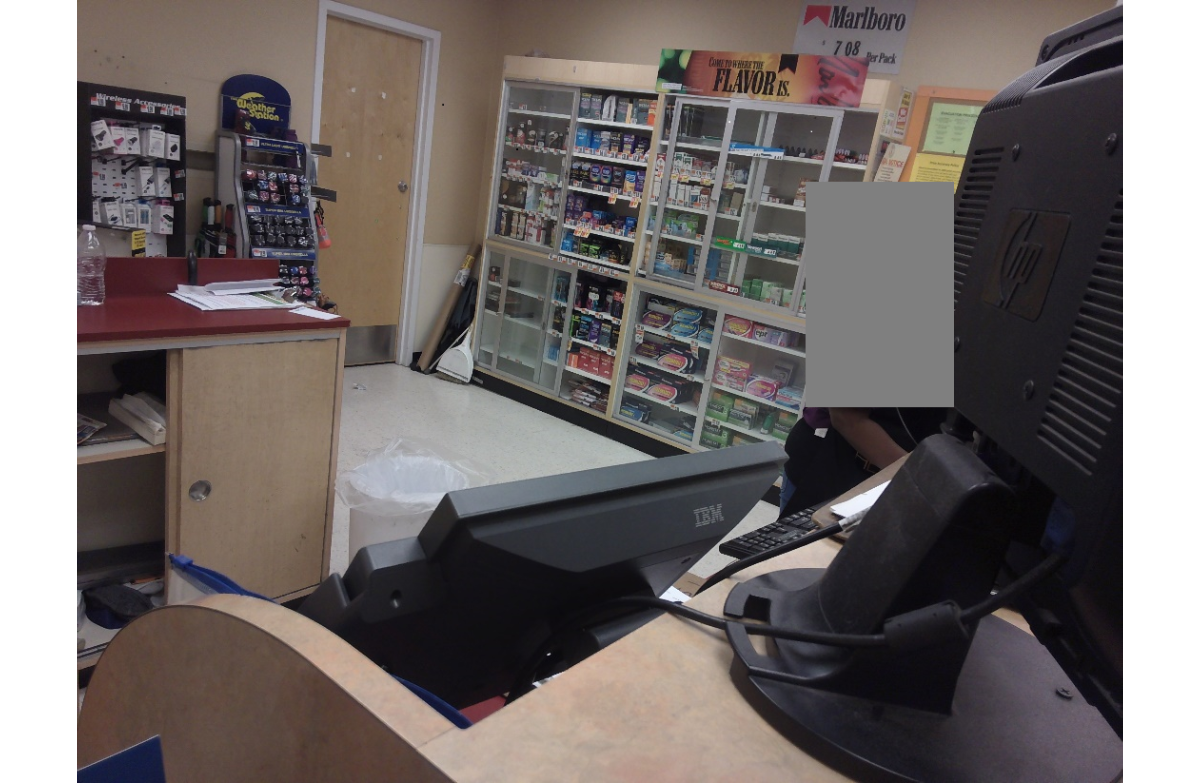
Panoramic image of a tobacco point of sale—view 2.

Our Inception V3 model fundamentally generated the predicted probabilities of the presence of each brand for each image in the validation set. Although we ultimately decided to focus on the brand Marlboro, we attempted to detect a series of brand logos. Success varied by brand, but our model achieved a classification accuracy of more than 75% for 8 of the 42 brands it was trained to detect (Textbox 1 and Table 1). For all but 7 brands—Camel, Marlboro, Pyramid, Pall Mall, Grizzly, Swisher, and Newport—the number of labeled example images constituted less than 11.7% (57/486) of the training data set. Table 2 shows the predicted probabilities of discovering specific logos in Figures 2 and 3 to illustrate the variability in the size and design of advertisements and the ability of the model to interpret branding in complex images. As shown, Newport was predicted with the highest probability in Figure 2, with Marlboro having the highest probability in Figure 3, with other brands still having high probabilities in each. Each image varies in terms of the heterogeneity and scale of the logos in question, implying the importance of both color and design.

**Tobacco brands considered for classification.**

**Cigarette**

- Marlboro
- Newport
- Camel
- Pall Mall
- Pyramid
- Maverick
- Santa Fe
- Winston
- Kool
- American Spirit

**Cigar**

- Swisher
- Black and Mild
- White Owl
- Dutch Masters
- Winchester
- Garcia y Vega
- Phillies
- Cheyenne
- Backwoods

**Smokeless tobacco**

- Levi Garrett Plug
- Day’s Work
- Red Man Plug
- Grizzly
- Garrett
- Skoal
- Red Man
- Copenhagen
- Red Seal
- Timberwolf
- Kayak
- Beechnut
- Kodiak
- Longhorn

**Snus**

- Skoal
- General

**e-Cigarettes**

- Blu
- FIN
- Logic
- MARKTEN
- NJOY
- V2
- VUSE

**Table 1:** Classification accuracy for validation data by tobacco brands.

Brand	Classification accuracy (%)
Copenhagen	90.4
Winston	85.1
Pyramid	82.7
Blu	81.7
American Spirit	80.3
Marlboro	78.8
Camel	75.9
Pall Mall	75.9

**Table 2:** Predicted probability of logos by Inception V3.

Photograph and brand			Probability
**Figure 2**			
	Marlboro	0.635	
	Newport	0.982	
	Camel	0.661	
**Figure 3**			
	Marlboro	0.993	
	Newport	0.868	

### Location of Advertisement Detection

**Figure 4:**
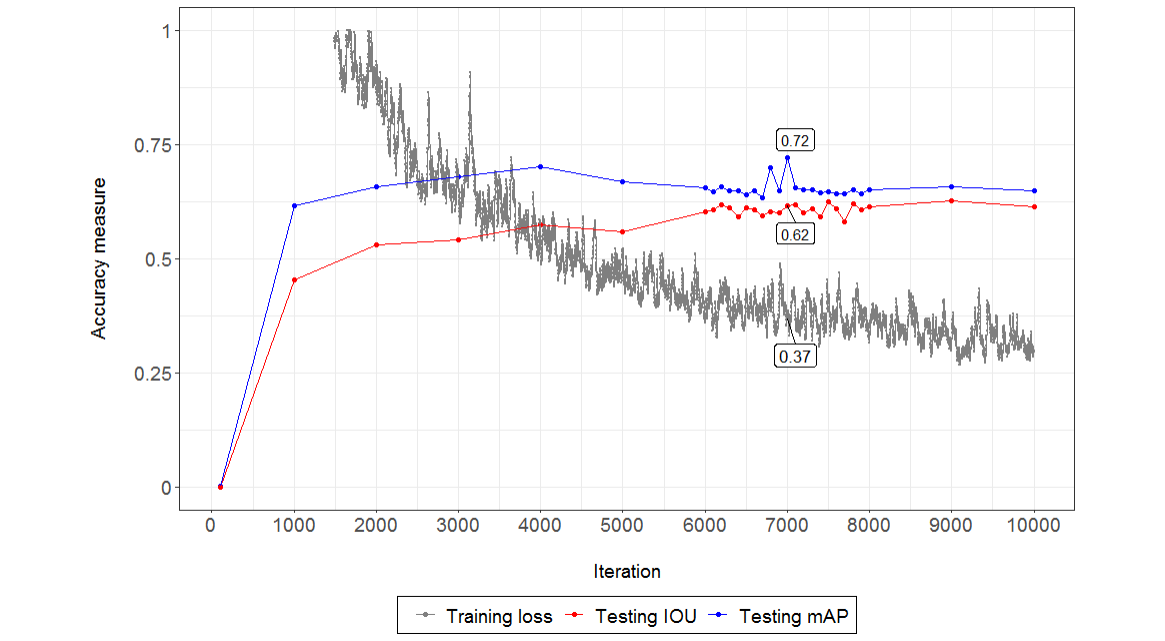
Training loss and testing accuracy of the YOLO (You Only Look Once) V3 objection detector. IOU: intersection over union; mAP: mean average precision.

**Figure 5:**
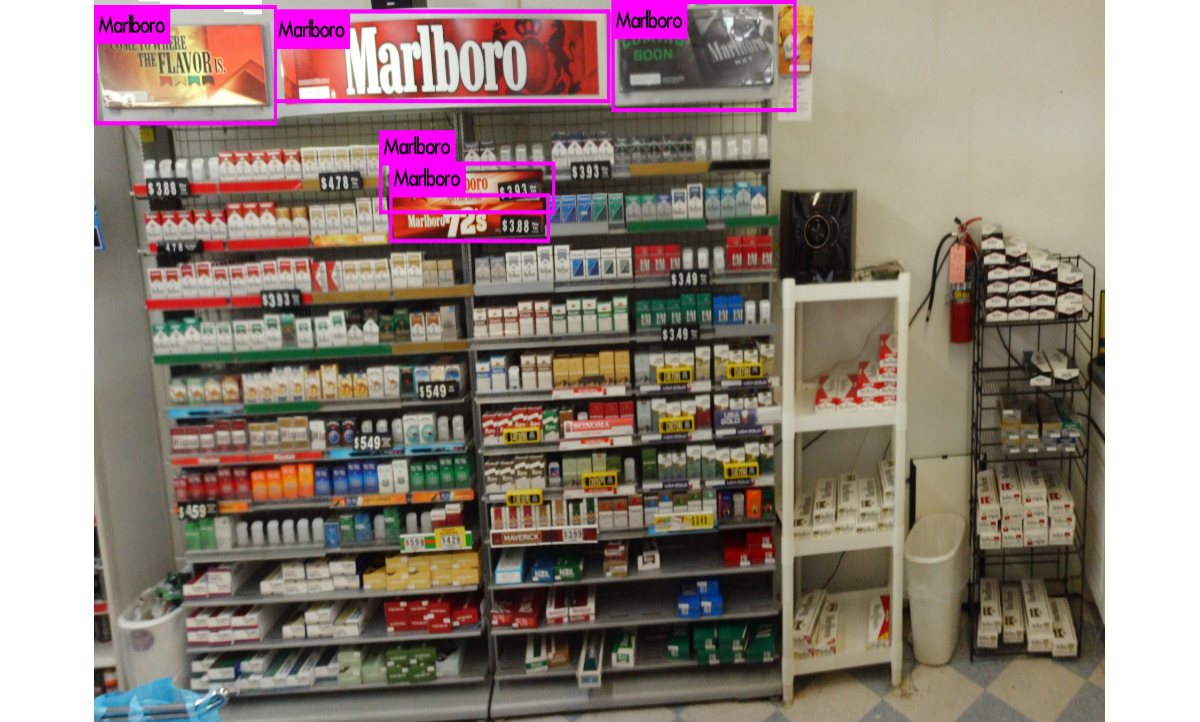
Prediction of Marlboro signs by YOLO (You Only Look Once) V3.

As described in the *Methods* section, for the purpose of location detection, we evaluated YOLO V3 model accuracy using the mAP and IOU on testing images. The network with weights that yielded the highest testing mAP (0.72; Figure 4) and IOU score (0.62; Figure 4) was chosen as the best model for detection. Figure 5 shows the detection results for an example image, where five Marlboro advertisements were detected. The object detector also generated a confidence score for each box, along with estimates of the upper left coordinates and the absolute width and height of the bounding boxes (Table 3).

**Table 3:** Bounding boxes of Marlboro signs detected by YOLO (You Only Look Once) V3.

Bounding box in Figure 5	Confidence score (%)	Bounding box measures (pixels)			
		Left x	Top y	Width	Height
1	100	0	16	737	376
2	100	724	30	1355	293
3	98	1149	505	708	163
4	100	1189	604	648	154
5	100	2084	0	745	358

## Discussion

### Principal Findings

Our study provides evidence of the feasibility of a novel methodological approach that tobacco researchers and other public health practitioners can apply in the collection and processing of data for tobacco and other POS surveillance efforts. Trained on a small set of labeled tobacco POS photographs, our classifier and object detector were able to identify tobacco brands and their location and dimension successfully within images. Although the initial labeling of the training data set was time-consuming, with an average of 3 minutes per photograph for staff to label advertisements, the costs of processing the photographic data decreased once the neural networks were trained. We were able to achieve a brand classification accuracy of over 75% for 8 of the 42 brands that our classifier (Inception V3) was trained to detect. Furthermore, we were able to accurately predict the location of branded advertising within the store environment (YOLO V3). Our location predictions achieved an mAP score of 0.72 and an IOU score of 0.62.

Our Inception V3 brand detection model had 2 major characteristics. First, the model was optimized to predict true positives, but with the unintended consequence of predicting more false positives. The model, therefore, tended to decide that a brand was in a photograph when none was present, in the process of finding true positives. Unfortunately, there was no alternative; tuning the model to favor predicting true negatives would have been too conservative (ie, unable to make any prediction on most photographs). Second, the model was heavily dependent on making predictions based on color. For example, in Figure 2, the size of the Marlboro advertisement is much larger than that in Figure 3. However, the predicted probability of a Marlboro advertisement in Figure 3 is higher owing to the presence of a familiar red logo, as shown in Table 1. The presence of green on the shelves of the menthol product displays may have caused the very high predicted probability of Newport in both photos, despite the Newport advertisements themselves being relatively small. Such variation in advertisement design and color, especially for Marlboro, may also explain why the accuracy of predicting the presence of Marlboro advertisements was among the lowest, despite having the most representation across photographs. However, our results indicate the potential for accurately identifying the presence of specific tobacco brands in digital photographs using Inception V3 or similar models. Although the accuracy rates shown above do not approach some in the medical field with very large training data sets, they do show how our approach is considerably more effective than random initialization constraints, even with a relatively small training set [27]. For context, a data set containing at least 2000 images of each brand with varying sizes, rotation angles, lighting schemes, and backgrounds would be needed for improved object detection accuracy [28].

This promising technology offers potential opportunities for tobacco POS surveillance to move forward. First, conducting timely, long-term surveillance of the POS environment can be resource intensive. Some existing state and local audit systems require a substantial amount of training and resources; however, they do provide critical information for enforcement activities as well as an understanding of the impact of marketing on current tobacco user behaviors and tobacco use initiation [1,3,13]. By applying machine learning techniques for efficient image classification and object detection, the methods described in this paper can assist in ensuring that retailers comply with specific retail provisions, such as state or local flavored tobacco bans, in addition to potentially improving the generalizability of research results by increasing the reliability and standardization of advertising information and classification.

The use of improved surveillance technologies can also assist in the assessment of local policy impacts in an evolving POS retail environment. With the adoption of local and state restrictions on the sale of flavored tobacco products, jurisdictions have an ever-growing need to assess the impact of these policies on tobacco retail environments [13]. For example, more routine and detailed retail POS advertising data could be used to assess differences before and after policies are implemented to examine key questions regarding changes in product availability, advertising, and promotion or discounting efforts.

Finally, this concept of applying machine learning to examine tobacco POS could also be extended to other public health applications. For example, image classification and object detection can be applied to identify other products of interest such as sugary drinks, candy, and processed foods from retail store images. Such an approach would allow public health researchers and policy makers to gauge the prevalence and types of advertisements for each product and understand how a specific retail food environment may interact with population demographics. Given the current attention on obesity, related health outcomes, and efforts to tax sugary drinks, we might anticipate interest in a machine learning–based approach, as illustrated [29].

### Limitations

Despite the success of applying deep neural networks to tobacco advertisement object detection, our analysis revealed several challenges, especially with respect to object location detection. Although our brand classification algorithms were generally successful (Inception V3) with some false-positive classifications, object location detection (YOLO V3) was more challenging, requiring a large amount of training data to identify patterns. Owing to the relatively small sample size, we decided to limit training our object detection algorithm to Marlboro advertisements, the dominant tobacco brand in our data set. Even when considering one brand within a given POS, we observe substantial variability in advertising content and appearance, including differences in color, shading, perspective, size, and rotation (Figure 5). Such variability made it difficult for object detection algorithms to learn and recognize brand patterns. Therefore, object location detection can be considered a more complex task prone to false negatives (ie, missing an object that is actually present). Our object detection model is conservative in its present form, with the side effect of missing true objects present in a photo, in contrast to our brand recognition model that generated false positives. Either bias would be improved by training the image classifier or object detector with additional images but would require further resources.

One key factor that limited our training data set is that relatively few tobacco POS advertising training images are available to the public, as distinct from generic computer vision applications where large and clean benchmark data sets exist. Collecting primary data on tobacco advertising and creating sufficient labeled training samples are a challenge in performing deep neural network–based object detection owing to time and cost implications. As shown in the literature, primary data collection for POS surveillance poses many challenges related to technology, workforce training, and overall logistics [16]. Data availability presents a particular concern for detecting tobacco brands that are less common in the marketplace, such as vape products, which are of increasing concern to researchers and health officials. Owing to the rapid evolution of branding, marketing, and delivery of tobacco products, we expect such analytical and data collection challenges to persist. Such challenges are further complicated by the lack of a centralized data store to track advertising materials. No comprehensive, publicly available, federal repository for tobacco advertising materials currently exists, although efforts by academic and nonprofit institutions to conduct marketing surveillance persist, including the Rutgers Center for Tobacco Studies and Campaign for Tobacco-Free Kids [30,31].

Beyond technology, there are also specific practical considerations when implementing such assessments. First, there are privacy and safety concerns associated with the use of camera glasses to collect POS photographs in public, especially when one or more identifying people are incidentally captured in photographs. Many store owners may not be comfortable with individuals using camera phones or handheld cameras to capture POS information. To accommodate this, camera glasses are often used to capture hands-free images in a more inconspicuous way. However, wearing a hidden camera in public may raise legal issues, depending on the location. For example, although capturing public scenes requires no consent in the United States, the opposite exists in Spain [32]. Although consent is not required in the United States for public photography, it may not always be socially acceptable. Clear guidelines about the “dos and don’ts” of wearing camera glasses for data collection should be created to broaden the applications of such technology to POS surveillance.

Finally, data quality must be considered when collecting data for machine learning and related analysis. As we discovered, cameras in glasses may not always capture high-quality or usable images. Photographs may be blurry, overexposed, or missing the desired images. As such, input data quality may be subject to bias and error, which could affect the development of subsequent machine learning algorithms. In addition, because many glasses do not have a remote trigger or a viewing screen, the individual using the glasses may be unaware of the image quality until the photos are uploaded. Such effects could result in a significant reduction in the availability of the training images used for image classification and object detection. However, having multiple overlapping images of any given POS location helps to ensure that all available POS advertising is captured with one or more clear photographs.

Future work on image classification in the tobacco POS retail environment would benefit from exploring additional methods to reduce false positives and false negatives for respective machine learning algorithms. For example, we could take advantage of image repositories that contain tobacco branding to help train models and add additional nuances. Although such repositories would not provide sufficient data to help describe the US retail environment comprehensively, they could assist by providing different geographical contexts or supplemental material in other advertising channels (eg, magazines, newspapers, films, the web, and social media) may still be helpful [33,34]. The introduction of crowdsourced data collection for use with machine learning techniques, as well as incorporating social media data, may also assist in gaining new sources of data and reducing the logistical burdens of surveillance programs. Integrating these efforts into a shared repository of tobacco-related images among tobacco control programs would also assist in improving brand identification and location detection and training of new algorithms. Such collaboration among local, state, federal, and academic institutions could be a powerful tool in understanding fast-changing retail trends and regional heterogeneity. In addition, our study considered location to be relative to each photograph, rather than in a defined geographic location within the POS. Although tobacco advertising tends to be consistent in the retail environment, such as in the commonly used Power Wall, value could be added by considering the purely geographic factor in future analyses [35].

### Conclusions

To summarize, our study demonstrated the utility of machine learning for POS assessment and highlighted some existing technological limitations. Although the current machine learning algorithms are advanced, they still have room for improvement. Large-scale POS photographic data sets that are comprehensive enough to capture a wider range of tobacco brands are needed to train multiclass algorithms capable of detecting advertisements from less common tobacco brands. Future studies should also attempt to detect the prices associated with within-store advertisements by incorporating text recognition algorithms to gain more contextual information on tobacco marketing. Attempting to classify an absolute geographic location within stores instead of relative image location within a photograph may also be of interest to researchers and surveillance efforts.

Despite these limitations, our analysis shows the promise of using machine learning as part of a suite of tools to understand the tobacco retail environment and inform public health interventions at multiple scales. The accurate and automatic classification of product brands and detection of their location within a retail environment could assist in developing a practical alternative to in-person POS audits, especially in resource-limited environments. For example, the necessary classifiers—documentation—could be made available in the public domain to facilitate their use by public health departments. In addition, coded photographs could be shared as part of a centralized resource to reduce the level of effort required to conduct or continue similar evaluations. With the increasing sales restrictions at the POS, surveillance products with enhanced contextual information about the retail environment can provide states, counties, and municipalities the opportunity to better understand the impact of existing and proposed policies, including ongoing efforts by the tobacco industry to target potentially vulnerable populations.

## Abbreviations

IOU: intersection over union
mAP: mean average precision
POS: point-of-sale
R-CNN: region-based convolutional neural networks
YOLO: You Only Look Once
